# Mechanical Properties of Clay-Reinforced Polyamide 6 Nanocomposite Liner Materials of Type IV Hydrogen Storage Vessels

**DOI:** 10.3390/nano14171385

**Published:** 2024-08-25

**Authors:** Dávid István Kis, Attila Bata, János Takács, Eszter Kókai

**Affiliations:** 1 Department of Automotive Technologies, Faculty of Transportation Engineering and Vehicle Engineering, Budapest University of Technology and Economics, Műegyetem rkp. 3, H-1111 Budapest, Hungary; kis.david@nje.hu (D.I.K.); takacs.janos@kjk.bme.hu (J.T.); 2 Department of Innovative Vehicles and Materials, GAMF Faculty of Mechanical Engineering and Computer Science, John von Neumann University, H-6000 Kecskemét, Hungary; bata.attila@nje.hu; 3 Department of Applied Sustainability, Széchenyi István University, Egyetem tér 1, 9026 Győr, Hungary

**Keywords:** hydrogen pressure vessel, polyamide 6, organoclay, nanocomposite, dynamic mechanical property

## Abstract

This study focuses on polyamide 6/organo-modified montmorillonite (PA6/OMMT) nanocomposites as potential liner materials, given the growing interest in enhancing the performance of type IV composite overwrapped hydrogen storage pressure vessels. The mechanical properties of PA6/OMMT composites with varying filler concentrations were investigated across a temperature range relevant to hydrogen storage conditions (−40 °C to +85 °C). Liner collapse, a critical issue caused by rapid gas discharge, was analyzed using an Ishikawa diagram to identify external and internal factors. Mechanical testing revealed that higher OMMT content generally increased stiffness, especially at elevated temperatures. The Young’s modulus and first yield strength exhibited non-linear temperature dependencies, with 1 wt. per cent OMMT content enhancing yield strength at all tested temperatures. Dynamic mechanical analysis (DMA) indicated that OMMT improves the storage modulus, suggesting effective filler dispersion, but it also reduces the toughness and heat resistance, as evidenced by lower glass transition temperatures. This study underscores the importance of optimizing OMMT content to balance mechanical performance and thermal stability for the practical application of PA6/OMMT nanocomposites in hydrogen storage pressure vessels.

## 1. Introduction

Type IV composite overwrapped pressure vessels have a polymer liner fully overwrapped by a fiber-reinforced composite structure. The liner ensures the tightness of hydrogen, and the composite shell withstands the inner pressure [1,2]. There is growing interest in the processing of polymer nanocomposites instead of neat polymers as liner materials for composite overwrapped pressure vessels (COPVs) to improve the safety of hydrogen fuel cell electric vehicles. One of the main gaps in knowledge is the examination of different filler concentrations of the most used liner polymers to acquire general knowledge of their effectiveness in practical working conditions of type IV hydrogen storage vessels (70 MPa, −40 °C; +85 °C). Most available studies on the permeability of polymer nanocomposites are regarding oxygen, water vapor, and carbon dioxide, as these gases are mainstream for the packaging industry [3,4]. Some studies already investigated polymer-layered silicate nanocomposites [5,6,7]. However, no comprehensive research that extensively studies the effect of a wide range of silicate content at a wide range of temperature with a focus on hydrogen storage has been found. In this paper, the mechanical properties of different polyamide 6/organo-modified montmorillonite (PA6/OMMT) nanocomposites are investigated as a prelude to subsequent hydrogen permeability research.

**Figure 1:**
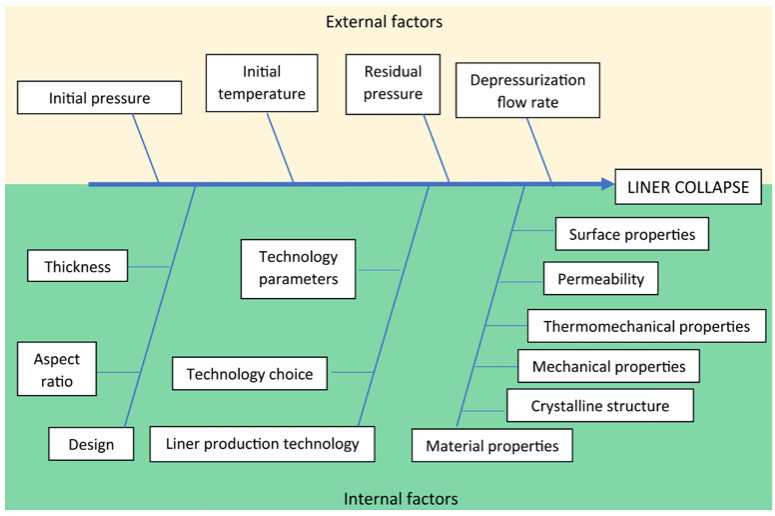
Ishikawa diagram with the effecting factors of liner collapse.

From a material compatibility point of view, the main issue of polymer liners is permeation, which is maximized by standards at 1.24·10^−15^ mol/(m·s·Pa) [6,8,9,10]. In a type IV hydrogen storage tank, if low hydrogen permeation is coupled with rapid gas discharge, liner collapse might happen. This phenomenon can be attributed to several variables that were identified in our previous review paper [11]. Once the pressure vessel is filled with hydrogen, the gas will permeate through the liner and the trapped hydrogen gas at the liner/overwrap interface will reach an equilibrium pressure. Depressurization results in a pressure gradient which can exceed the stress limit defined by the permeation and thermomechanical limitations of the liner material. A decompression rate greater than the tolerable limit results in liner collapse [11]. The factors leading to liner collapse are multicomponent and can be divided into two groups: external and internal factors. Figure 1 presents external factors (such as the depressurization flow rate and the initial pressure) and internal factors (those that can be influenced by liner design, production technology, and material properties).

Polyamide 6 (PA6) is a popular technical polymer in liner production, but the major application focus of PA6 nanocomposites is in high-barrier packaging [12]. Polyamides are polar, with molecules creating strong intermolecular interactions. This results in a semicrystalline structure, and the tightly packed polymer chains create diffusion barriers for hydrogen molecules. The free volume and polymer segmental mobility of PA6 depend on its crystallinity. Higher crystallinity means less free volume, decreased segmental mobility, and lower gas permeability and it can be affected by several factors, such as the type of polymer, the processing conditions, and the presence of additives [5,6,7,13]. Since the decompression of COPVs involves the cooling of the medium, PA6 has an advantage over the also common polyethylene liner materials, as PA6 is likely to be subjected to a pressure gradient in its glassy state during this process. This makes PA6 a more durable liner choice compared to HDPE [2,5,6,11].

The addition of lamellar clay minerals in polymers is reported to be effective in the improvement of the tensile modulus, mechanical strength, temperature resistance, and the decrease in gas permeability [14,15]. Fillers can decrease free volume and increase glass transition temperature (T_g_) and crystallinity due to the nucleating effect of nanoparticles.

By examining PA6 composites with varying OMMT filler concentrations across a broad temperature range (−40 °C to +85 °C), this study seeks to address the critical issue of liner collapse during rapid gas discharge. Utilizing mechanical testing and dynamic mechanical analysis, the investigation reveals the effects of OMMT content on material brittleness, Young’s modulus, yield strength, and thermal stability. The findings highlight the dual role of OMMT as both a reinforcing agent and a potential detriment to toughness and heat resistance, underscoring the need for the precise optimization of OMMT content to enhance the performance and reliability of hydrogen storage liners.

## 2. Materials and Methods

### 2.1. Sample Preparation

Neat polyamide 6 powder, Alphalon® 24 (Grupa Azoty S.A., Tarnów, Poland), was used in this research study. It is intended for rotomolding, which makes it suitable for hydrogen COPV liner production. The montmorillonite organoclay was Nanomer® I.34TCN (Nanocor Inc., Arlington Heights, IL, USA) with an organic surfactant of methyl octadecyl bis-2-hydroxyethyl ammonium methyl sulphate. Filler concentrations of PA6/OMMT nanocomposites were chosen to be 1, 2.5, 5, and 10 wt. per cent. In fact, the bare montmorillonite content was different, because the surface modifier concentration in OMMT was 30–32 wt. per cent according to the technical data sheet of the clay.

The composites were developed using an extrusion–injection process: (1) Weigh an appropriate amount of OMMT. (2) Shake the composite powder in a sealed bucket to create a uniform mixture. (3) Melt compound the neat and composite materials with a LHFS1-271022-type twin crew extruder (Labtech Ltd., Hopkinton, MA, USA). (4) Granulate the extruded filaments to a uniform size. Both the neat PA6 and PA6/OMMT nanocomposites were dried at 80 °C for 6 h. Then, the fully dried material was used to create dog bone-shaped specimens with an e-mac 80 type injection molding machine (Engel GmbH., Schwertberg, Austria). The injection-molded specimens refer to the 1A sample of ISO 527 standard with the length, width, and thickness of 80 mm, 10 mm, and 4 mm, respectively. Both neat PA6 and PA6/OMMT nanocomposite specimens were conditioned for 1 week prior to testing (23 °C +/− 2 °C, 50 per cent +/− 10 per cent humidity).

### 2.2. Tensile Test

The static tensile tests were performed at 4 different temperatures in the working temperature range of hydrogen COPVs according to UN Regulation No. 134: −40 °C, 0 °C, 30 °C, 85 °C [9,16]. The mechanical properties of neat PA6 and PA6/OMMT nanocomposites are discussed in the literature [17,18], but to our knowledge, this is the first investigation of the relationship between tensile properties, clay content, and temperature at the same time. The measurements were performed by a 3366 type (Instron Inc., Norwood, MA, USA) universal testing machine. All temperature ranges were maintained in a 3119-409 type climate chamber (Instron Inc., USA) during the tests, with liquid nitrogen used as the refrigerant to achieve −40 °C. Crosshead speed was 10 mm/min. Every measurement was repeated on five specimens from separate samples.

### 2.3. Dynamic Mechanical Analysis

Dynamic mechanical analysis (DMA) is especially important from the perspective of hydrogen barrier properties because of the existing correlation between transport phenomena and dynamic mechanical properties. DMA tests were performed on a DMA Q800 type (TA Instruments Inc., New Castle, DE, USA) measuring device in dual cantilever operational mode with a strain amplitude of 15 microns and a frequency of 1 Hz. The scanning temperature ranged from −90 °C to 150 °C at a heating rate of 2 °C/min. The test pieces were cut from the injection-molded dog bone specimen to a length, width, and thickness of 60 mm, 10 mm, and 4 mm, respectively.

## 3. Results and Discussion

### 3.1. Mechanical Properties

**Figure 2:**
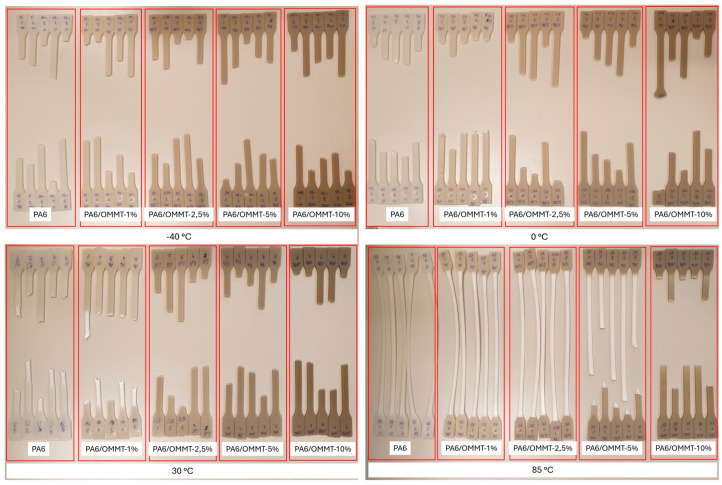
Tensile specimens after testing between −40 °C and 85 °C.

A fundamental question is addressed in this research study, namely, how the temperature and OMMT composition affect the yielding deformation of PA6 at the temperature range of hydrogen storage tanks. The visual inspection of the tested specimens in Figure 2 demonstrates the combined effect of temperature and clay content. Lower temperatures (−40 °C and 0 °C) result in more brittle fractures across all specimens. As temperature increases (30 °C and 85 °C), ductility generally increases, except for higher clay content at specific temperatures. OMMT content at 10 wt. per cent shows brittle behavior at all temperatures, while lower OMMT content (one per cent) shows similar ductility compared with neat PA6. The variation in fracture appearance among the five specimens for each test condition is due to inherent material heterogeneity and minor differences in processing and alignment, which can cause slight deviations in fracture behavior, even under identical testing conditions.

**Figure 3:**
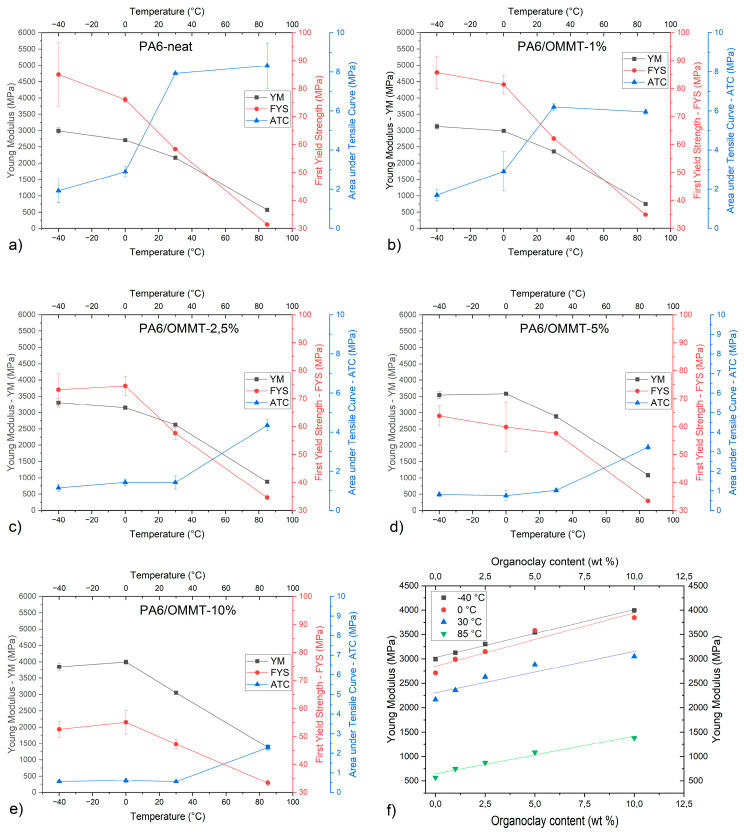
Young’s modulus, first yield strength, and area under tensile curve vs. temperature values of (**a**) neat PA6; (**b**) PA6/OMMT-1 per cent; (**c**) PA6/OMMT-2,5 per cent; (**d**) PA6/OMMT-5 per cent; (**e**) PA6/OMMT-10 per cent; and (**f**) Young’s modulus plotted against clay content.

Figure 3 shows the Young’s modulus, the first yield stress, and toughness as a function of temperature. The method used for measuring yield stress is based on the intersecting lines from the initial modulus and the almost linear region past the yield point. The toughness was evaluated by integrating the area under the tensile curve up to the first yield stress across different temperatures.

Unexpectedly, the results indicate that the ductility of neat PA6 and PA6/OMMT composites decreases more rapidly at higher temperatures, suggesting a non-linear relationship between temperature and Young’s modulus, deviating from the typically expected linear behavior (Figure 3a–e) [17]. The same observation is true for first yield strength as well. The linear dependence of the modulus and OMMT content is in agreement with the literature (Figure 3f) [19]. A new observation was made about the dependence of first yield strength and clay content. The first yield strength of all PA6/OMMT-1 per cent specimen outperforms neat PA6 at every temperature. Considering the high standard deviation of first yield strength values at −40 °C and 0 °C, PA/OMMT-2,5 per cent shows minimal differences from the neat PA6. Furthermore, the yield strength of all composites outperforms neat PA6 at 85 °C, which confirms the role of the clay in the structural development of nanocomposites.

At a given temperature, the increase in OMMT content results in lower toughness, which is consistent with the literature [14]. Bureau et al. [20] suggested that the reinforcing effect plays an important role in rigidity. It is worth noting that among all composites, the PA/OMMT-1 per cent composition has a negligible decrease in toughness compared to neat PA6 between −40 °C and 30 °C.

### 3.2. Dynamic Mechanical Properties

The stiffness of the neat PA6 and its composites has been analyzed between −90 and 150 °C, considering the effect of layered particles on heat resistance of PA6. In this temperature range, PA6 composites exhibit two dynamic mechanical loss maxima: primary (α relaxation) and secondary (β relaxation) in the ranges of 50–75 °C and −70 to −50 °C, respectively [17,19,21,22]. β relaxation is related to the movement of polar groups that are not hydrogen bonded, while α relaxation is associated with the glass transition where the long chains of the amorphous region gain mobility [21,23].

**Figure 4:**
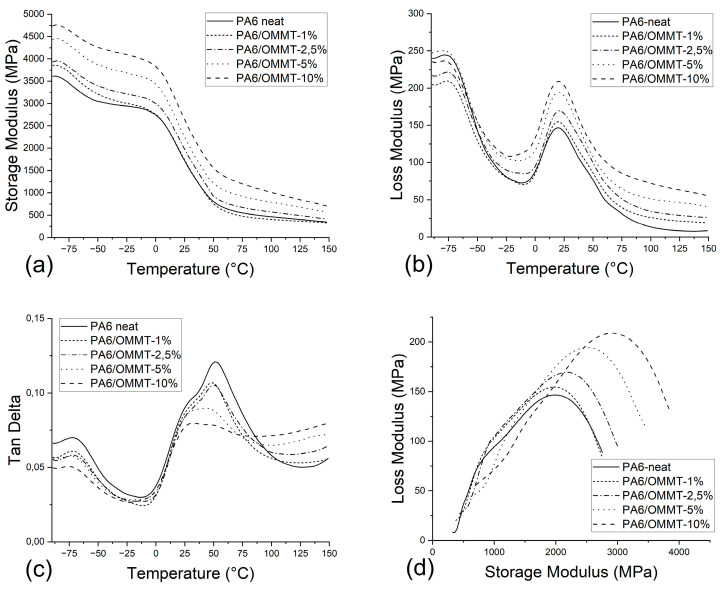
Results by DMA measurement of neat PA6 and PA6/OMMT composites: (**a**) storage modulus, (**b**) loss modulus, (**c**) damping as a function of temperature, (**d**) Cole–Cole plot of loss modulus vs. storage modulus.

As depicted in Figure 4a, the storage modulus presents an increasing trend with increasing content of OMMT. At 30 °C the storage moduli of neat PA6, PA6/OMMT-1 per cent, PA6/OMMT-2,5 per cent, PA6/OMMT-5 per cent, and PA6/OMMT-10 per cent were 1495 MPa, 1509 MPa, 1745 MPa, 2001 MPa, and 2376 MPa, respectively. The general increase in storage modulus with filler content is observed in other PA6 composite configurations, as well [17,19,21,22]. The improvement in storage modulus is presumably due to the fine dispersion of the clay particles [21].

The stiffening effect of OMMT is visible on the loss modulus–temperature charts in Figure 4b. Generally, the loss modulus values at the α relaxation peaks of composites are above that of neat PA6, meaning higher energy dissipation during glass transition. This could be due to the interaction between the polymer matrix and the OMMT nanoparticles, which restricts the mobility of the polymer chains. It is also observed that a clay content of 5 wt. per cent or above results in near elimination of the β relaxation peak. The composition of less intense β relaxation peaks can be expected to be brittle [23]. These indicate decreased toughness in these composites, which is confirmed by the area values under the tensile curves [23]. 

Tan δ versus temperature is plotted in Figure 4c. The peak at −75 °C, which is related to β relaxation, remains visible for every composition. The tan δ curve has a peak around 50 °C related to α relaxation, with a shoulder peak at 25 °C. The intensity of the former decreases with the increase in clay content, indicating a reduction in the amount of α relaxation. This may also indicate a decrease in the number of amorphous molecules. It is important to note that the occurrence of the shoulder peaks of the PA6/OMMT-1 per cent and PA6/OMMT-2,5 per cent compositions exhibit similarities with neat PA6, which reflects improved yielding behavior thanks to the synergetic effect of the deformation of amorphous and crystalline phases [17]. It can be seen how this shoulder grows dominant over the 50 °C peak at higher OMMT concentrations, which observation correlates with the first yield strength trends acquired from tensile tests. The deterioration in yielding behavior of PA6/OMMT-5 per cent and above seems to be originated from the blocking of the amorphous regions caused by the increase in filler.

In the Cole–Cole diagram, the components of the complex modulus recorded in the temperature range of α relaxation are plotted against each other (Figure 4d). These diagrams are particularly useful for examining multiphase systems like polymer blends and composites [24]. Homogeneous polymer systems typically exhibit a semicircle. Nanoclay composites deviate from a semicircular shape, implying that the system is heterogeneous [25]. If there are no structural changes due to incorporation of nanofiller, the Cole–Cole plot for the nanocomposite would superimpose upon that of the neat PA6. As depicted in Figure 4d, the unfilled PA6 is also a distorted semicircle, which can be explained by the semicrystalline morphology (crystalline–amorphous). All composites indicate structural changes, being more prominent at 5 and 10 wt. per cent OMMT content.

**Figure 5:**
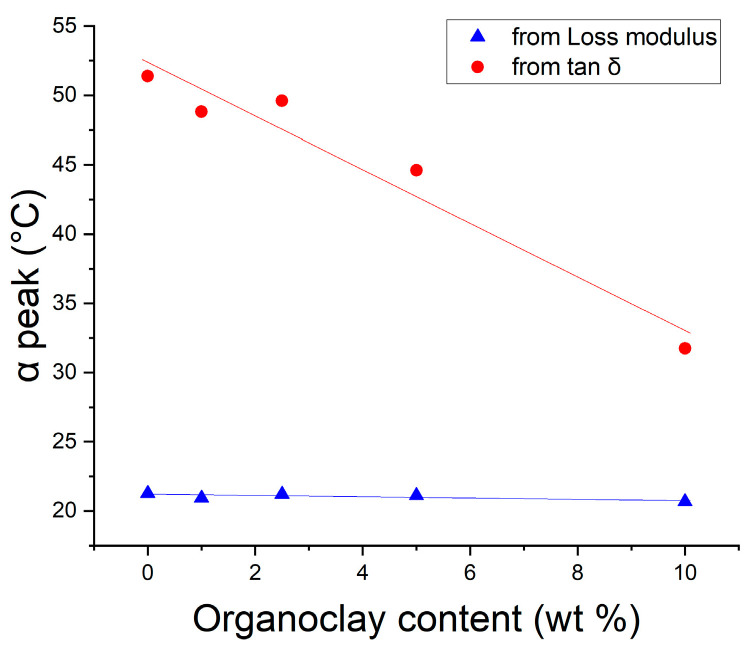
Tg originated from the α relaxation peak of loss modulus and tan δ curves.

T_g_ is referred to as one of the main physical parameters used to evaluate the heat distortion temperature of polymers and composites. Although the T_g_ value can be easily identified by the sharp decrease in storage modulus, the way the T_g_ is chosen often leads to confusion. While tan δ peak is often used to identify T_g_, loss modulus maximum is the more appropriate value, as it is the onset “softening” point related to the initiation of long-chain segmental motion [23]. Figure 5 shows that the increase in OMMT lowers the difference between T_g_ values concluded from tan δ and loss modulus peaks. It can also be concluded that the T_g_ of tan δ maximum is more sensitive to filler content than that of loss modulus.

As Pramoda et al. [21] showed, dried PA6 specimens exhibit minor differences in tan δ and loss modulus peaks. Because our specimens were conditioned for 1 week in 50 per cent humidity, the difference was much more significant in our case. The mentioned literature [21] also pointed out that the increasing moisture content causes a drastic decrease in loss modulus maxima values, thus making it confusing to determine T_g_. 

## 4. Conclusions

The major objective of this work is to establish the relationship between the complex mechanical properties of PA6/OMMT nanocomposite liner materials and the working temperature range of type IV hydrogen storage tanks. It can be stated, based on the observations, that with a growing amount of nanoparticles, Young’s modulus keeps on increasing, while impact strength decreases. A clay content of 1 wt. per cent shows a positive effect on yield stress at all investigated temperatures, and clay content generally enhances yield strength at 85 °C. Measurements over a wide temperature range revealed that the temperature sensitivity of Young’s modulus and the first yield stress is not linear, despite the literature data.

The improved yielding behavior of 1 wt. per cent OMMT content nanocomposite is confirmed by DMA measurements. DMA results also show that OMMT helps to improve the storage modulus of PA6/OMMT composites, which suggests a fine dispersion of filler particles. It was observed that the well-dispersed OMMT nanoparticles create a more rigid and less mobile network within the PA6 matrix. OMMT is found to lower T_g_, increase storage and tensile modulus, and lower impact strength. The proper investigation of T_g_ is found to be crucial in the special use case of hydrogen storage pressure vessels, as clay content can adversely affect the softening of the PA/OMMT nanocomposites.

In the context of hydrogen storage, these mechanical properties are particularly relevant as they directly impact the liner material’s performance under the conditions of liner collapse, as was presented in the Ishikawa diagram. The stiffness provided by the 1 wt. per cent OMMT content is favorable, but care must be taken to avoid excessive rigidity, which could compromise the tank’s safety and durability. The results indicate that higher clay content may lead to materials that are too brittle, posing a risk during the manufacturing and operational phases of the tanks. Therefore, the 1 wt. per cent clay loading appears to be an optimal balance, offering enhanced mechanical properties without introducing excessive brittleness. This balance is critical for ensuring the structural integrity and longevity of hydrogen storage systems under real-world conditions.

The mechanical and DMA investigations conducted in this study have provided a preliminary screening to identify the most promising material blends. Further studies, including thermal and permeability analyses, are necessary to fully characterize these materials and optimize them for their intended application.

## Data Availability

The data presented in this study are available on request from the corresponding author.
